# Role of the Na^+^/H^+^ exchanger 3 in angiotensin II-induced hypertension in NHE3-deficient mice with transgenic rescue of NHE3 in small intestines

**DOI:** 10.14814/phy2.12605

**Published:** 2015-11-12

**Authors:** Xiao C Li, Gary E Shull, Elisa Miguel-Qin, Fang Chen, Jia L Zhuo

**Affiliations:** 1 Laboratory of Receptor and Signal Transduction, Department of Pharmacology and Toxicology, Division of Nephrology, Department of Medicine, University of Mississippi Medical CenterJackson, Mississippi; 2 Department of Molecular Genetics, University of Cincinnati College of MedicineCincinnati, Ohio

**Keywords:** Angiotensin II, hypertension, intestines, kidney, NHE3

## Abstract

The role of Na^+/^H^+^ exchanger 3 (NHE3) in the kidney in angiotensin II (ANG II)-induced hypertension remains unknown. The present study used global NHE3-deficient mice with transgenic rescue of the *Nhe3* gene in small intestines (tg*Nhe3*^*−/−*^) to test the hypothesis that genetic deletion of NHE3 selectively in the kidney attenuates ANG II-induced hypertension. Six groups of wild-type (tg*Nhe3*^*+/+*^) and tg*Nhe3*^*−/−*^ mice were infused with either vehicle or ANG II (1.5 mg/kg/day, i.p., 2 weeks, or 10 nmol/min, i.v., 30 min), treated with or without losartan (20 mg/kg/day, p.o.) for 2 weeks. Basal systolic blood pressure (SBP) and mean intra-arterial blood pressure (MAP) were significantly lower in tg*Nhe3*^*−/−*^ mice (*P *<* *0.01). Basal glomerular filtration rate, 24 h urine excretion, urinary Na^+^ excretion, urinary K^+^ excretion, and urinary Cl^−^ excretion were significantly lower in tg*Nhe3*^*−/−*^ mice (*P *<* *0.01). These responses were associated with significantly elevated plasma ANG II and aldosterone levels, and marked upregulation in aquaporin 1, the Na^+^/HCO_3_ cotransporter, the *α*1 subunit isoform of Na^+^/K^+^-ATPase, protein kinase C*α*, MAP kinases ERK1/2, and glycogen synthase kinase 3 *α*/*β* in the renal cortex of tg*Nhe3*^*−/−*^ mice (*P *<* *0.01). ANG II infusion markedly increased SBP and MAP and renal cortical transporter and signaling proteins in tg*Nhe3*^*+/+*^, as expected, but all of these responses to ANG II were attenuated in tg*Nhe3*^*−/−*^ mice (*P *<* *0.01). These results suggest that NHE3 in the kidney is necessary for maintaining normal blood pressure and fully developing ANG II-dependent hypertension.

## Introduction

Hypertension is well recognized as a chronic medical condition, affecting approximately one in three adults worldwide (Kearney et al. 2005; Lloyd-Jones et al. 2010). It has been estimated that up to 90–95% of patients with this medical condition have essential hypertension without clearly identified causes, whereas the remaining 5–10% have secondary hypertension with known genetic or endocrine disorders (Kearney et al. 2005; Lloyd-Jones et al. 2010). Although the etiologies and underlying mechanisms of essential hypertension remain incompletely understood, genetic, endocrine, dietary and environmental, and central neural factors have all been extensively investigated. For example, several genome-wide association studies (GWAS) have recently identified over 50 common genetic variants or loci associated with blood pressure or hypertension in humans, however the contribution of each of these common genetic variants to the development of hypertension is much smaller than expected (Levy et al. 2009; Newton-Cheh et al. 2009; Ehret 2010; Kato et al. 2011). Likewise, the role of high salt intake in the development hypertension has also been extensively investigated in salt-sensitive animal models, but 8% salt and deoxycorticosterone acetate (DOCA) are too often administered to induce hypertension. The relevancy of this model to human essential hypertension remains unknown, given that the U.S. Dietary Guidelines for Americans recommend eating a diet of 100 mmol of sodium a day or lower (Sacks et al. 2001; Chobanian et al. 2003; McCarron 2003). Furthermore, the role of increased renal sympathetic nerve activity in hypertension has also been extensively studied, but one of the most rigorously designed and randomized studies, the SYMPLICITY HTN-3 (Renal Denervation in Patients With Uncontrolled Hypertension) trial, has failed to show significant antihypertensive effect with catheter-based renal artery denervation (Bakris et al. 2015). Clearly, more studies are required to further uncover the mechanisms of essential hypertension.

Regardless of the precise and/or direct cause or factor, the development, progression, and maintenance of most, if not all, forms of hypertension appear to converge on a final common pathway, increased salt reabsorption or retention due to abnormal renal sodium (Na^+^) handling involved with or without ANG II (Guyton 1991; Guyton et al. 1995). In the kidney, over 70% of the filtered Na^+^ load is reabsorbed by the proximal nephron (Rector 1983; Weinstein 2008; Zhuo and Li 2013). Among all Na^+^ transporters or cotransporters in different nephron segments, the sodium and hydrogen exchanger 3, NHE3, is considered to be the most important Na^+^ transporter in apical membranes of the proximal tubule and loop of Henle (Lorenz et al. 1999; Wang et al. 1999; Vallon et al. 2000; McDonough 2010). NHE3 acts to secrete H^+^ from the cells in exchange for luminal Na^+^ entry, directly and indirectly contributing to up to 75% of Na^+^ and 90% of 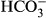 reabsorption in the proximal tubule Na^+^ reabsorption and body acid–base balance (Aronson 1983; Rector 1983; Boron and Boulpaep 1989). It is therefore not difficult to extrapolate from the function of NHE3 that it may play a potential role in mediating sodium retention and hypertension in humans. However, the role of NHE3 in the kidney, especially in the proximal tubule, in the development of angiotensin II (ANG II)-dependent hypertension has not been investigated previously. Given that ANG II plays an important role in increasing the expression of NHE3 or activity in the proximal tubule (Houillier et al. 1996; Wang et al. 1999; Banday and Lokhandwala 2011; Li and Zhuo 2011), the present study test the hypotheses that NHE3 in the kidney is necessary for maintaining long-term blood pressure homeostasis, and that genetic deletion of NHE3 in the kidney attenuates the development of ANG II-induced hypertension in mice.

## Methods

### Animals

Heterozygous breeding pairs of NHE3 mutant mice with transgenic rescue of the *Nhe3* gene in small intestines (tg*Nhe3*^*+/−*^) were generously provided by Dr. Gary E. Shull of the University of Cincinnati College of Medicine (Woo et al. 2003; Noonan et al. 2005). Homozygous mutant tg*Nhe3*^*−*/−^ mice were generated by breeding tg*Nhe3*^*+/−*^ mice and genotyped in this laboratory according to Woo et al. (2003) and Noonan et al. (2005), respectively. Specifically, genotyping was carried out using the 5′-oligonucleotide primer sequence from the intestinal fatty acid binding protein (IFABP) promoter sequence (5′-CTGCCAGGTTATCTCTTGAAC-3′), and the 3′ reverse primer sequence from the NHE3 cDNA sequence (5′-CTGTTCGGTTCCTCCTCAATG-3′). PCR conditions for genotyping were set as: 94°C for 3 min, then 35 cycles at 94°C for 30 sec, 60°C for 30 sec, and 72°C for 30 sec, followed by 72°C for 10 min (Woo et al. 2003; Noonan et al. 2005). All tg*Nhe3*^−/−^ mice used in the present study carried the IFABP/NHE3 transgene in small intestines and had deletion of the *Nhe3* gene primarily in the kidney (Woo et al. 2003; Noonan et al. 2005). Because very little NHE3 is expressed outside the digestive system and the kidney, tg*Nhe3*^*−/−*^ mice were used as an alternative kidney-selective NHE3-KO mouse model, whereas age-matched tg*Nhe3*^*+/+*^ littlemates were used as wild-type controls. The experiments as described in this study were approved by the Institutional Animal Care and Use Committee of the University of Mississippi Medical Center.

### Angiotensin II-induced hypertension in tg*Nhe3*^*+/+*^ and tg*Nhe3*^−/−^ mice

Induction of ANG II-induced hypertension in tg*Nhe3*^*+/+*^ and tg*Nhe3*^*−/−*^ mice was performed as we described previously (Zhuo et al. 2002; Li et al. 2007; Li and Zhuo 2011, 2013). Briefly, three groups of adult male tg*Nhe3*^*+/+*^ and tg*Nhe3*^*−/−*^ mice (*n* = 10–15 each group) were anesthetized with pentobarbital (50 mg/kg, i.p.) and implanted with an osmotic minipump (Model 2002) to infuse vehicle saline or a high pressor dose of ANG II (Bachem., Torrance, CA, 1.5 mg/kg/day, i.p.). Mice in Group 1 received saline and served as time controls. Mice in Group 2 were infused with ANG II for 2 weeks (Li et al. 2007; Li and Zhuo 2013). Mice in Group 3 were infused with ANG II as in Group 2 and concurrently treated with the AT_1_ receptor blocker losartan (20 mg/kg/day, p.o.) for 2 weeks. Our previous studies have shown that at this dose of ANG II, blood pressure rose robustly and progressively in mice over the 2-week period (Li et al. 2007; Li and Zhuo 2008a, 2013). Thus, the tail-cuff technique was used to determine basal and weekly systolic blood pressure responses to ANG II or losartan (Zhuo et al. 2002; Li et al. 2007; Li and Zhuo 2008a). Additionally, 24 h fecal and urine samples were collected using metabolic cages to determine fecal and urinary Na^+^ excretory responses in tg*Nhe3*^*+/+*^ and tg*Nhe3*^*−/−*^ mice before and after ANG II was infused (Zhuo et al. 2002; Li et al. 2007; Li and Zhuo 2008a). Plasma, urinary, and fecal Na^+^, K^+^, and Cl^−^concentrations were determined by NOVA 13 Electrolyte Analyzer (Nova Biomedical, Waltham, MA).

### Pressor responses to ANG II in anesthetized tg*Nhe3*^*+/+*^ and tg*Nhe3*^−/−^ mice

To further confirm the differences in basal systolic blood pressure and its responses to ANG II in conscious tg*Nhe3*^*+/+*^ and tg*Nhe3*^−/−^ mice, three additional groups of tg*Nhe3*^*+/+*^ and tg*Nhe3*^*−/−*^ mice (*n* = 6–8 each group) were anesthetized with Inactin (50 mg/kg wt., i.p.). The left carotid artery and jugular vein were cannulated with a polyethylene tube (PE50) with a fine fabricated tip to measure mean intra-arterial blood pressure (MAP) using a PowerLab Data Acquisition System (ADInstruments, Colorado Springs, CO) and to infuse saline containing 3% FITC-inulin to measure the whole kidney glomerular filtration rate (GFR) and 2% BSA (10 *μ*L/min, i.v.) for 30 min before ANG II (10 pmol/min, i.v.) was infused for additional 30 min, as we described previously (Li and Zhuo 2008a; Li et al. 2009, 2012b). Mice in Group 1 served as time controls. Mice in Group 2 received ANG II infusion only, whereas mice in Group 3 were pretreated with the AT_1_ receptor blocker losartan (20 mg/kg, p.o, 3 days) before ANG II was infused to determine the role of the AT_1_ receptor.

### Measurement of plasma ANG II and aldosterone levels in tg*Nhe3*^*+/+*^ and tg*Nhe3*^−/−^ mice

Upon the completion of the experiment, blood samples were collected from tg*Nhe3*^*+/+*^ and tg*Nhe3*^−/−^ mice upon decapitation in an inhibitor cocktail solution for measurement of plasma ANG II and aldosterone concentrations, as described previously (Zhuo et al. 2002; Li et al. 2007; Li and Zhuo 2011). Plasma samples were immediately extracted for measurement of plasma ANG II and aldosterone levels using a sensitive aldosterone (Cat. 501090; Cayman, Ann Arbor, MI) or ANG II enzyme immunoassay kit (Cat. S-1133; Bachem.) (Li et al. 2007; Li and Zhuo 2008a, 2011), respectively. Aldosterone and ANG II concentrations are expressed as pg/mL.

### Molecular, morphological, and reabsorptive phenotypes of the intestines and the kidney in tg*Nhe3*^*+/+*^ and tg*Nhe3*^−/−^ mice

To determine the intestinal and kidney phenotypes, the entire digestive system and the kidneys were collected from age- and body weight-matched male tg*Nhe3*^*+/+*^ and tg*Nhe3*^*−/−*^ mice for comparisons of NHE3 mRNA and protein expression in the renal cortex, net weights, histology, and absorptive function, as described previously (Woo et al. 2003; Noonan et al. 2005). Briefly, the guts and kidneys were blotted dry and weighed, whereas fluid accumulation within the intestines, largely in the cecum, was collected from tg*Nhe3*^*+/+*^ and tg*Nhe3*^*−/−*^ mice and Na^+^, K^+^, and Cl^−^ concentrations measured accordingly (Woo et al. 2003; Noonan et al. 2005). The kidneys were sectioned and stained by Masson Trichrome for histological examination of the glomeruli and proximal tubule structures (Li et al. 2009; Li and Zhuo 2011).

### Western blot analysis of renal cortical major Na^+^ cotransporters, water channel aquaporin 1 protein, or signaling proteins and their responses to ANG II in tg*Nhe3*^*+/+*^ and tg*Nhe3*^*−/−*^ mice

The expression of some major Na^+^ cotransporters and water channel aquaporin 1 protein in the renal cortex of tg*Nhe3*^*+/+*^ and tg*Nhe3*^*−/−*^ mice, primarily in the proximal tubules, as well as major signaling protein responses at basal levels and during ANG II infusion was determined using western blot analysis (Li and Zhuo 2011, 2013; Li et al. 2012a). These included the Na^+^/HCO_3_ cotransporter (NBC), the *α*1 subunit isoform of Na^+^/K^+^-ATPase, aquaporin 1 (AQP1), protein kinase C*α* (PKC*α*), MAP kinases ERK1/2, and glycogen synthase kinase 3 *α*/*β* (GSK3*α*/*β*) (Li and Zhuo 2011). These transporters and signaling proteins play an important role in regulating proximal tubule Na^+^ transport and respond to ANG II stimulation (Li and Zhuo 2011, 2013; Li et al. 2012a). For western blot analysis, the following antibodies were used: rabbit polyclonal anti-Na^+^/HCO_3_ cotransporter (NBC) targeting the N-terminus 338–391 of the rat kidney Na^+^/HCO_3_ cotransporter (Abcam, Cambridge, MA, Cat. #AB3212), the mouse monoclonal anti-Na^+^/K^+^-ATPase recognizing the *α*1 subunit isoform of Na^+^/K^+^-ATPase (Millipore Cat., Billerica, MA, #05-369; Lot: #DAM1794271), the rabbit polyclonal antiprotein kinase C*α* antibody targeting phospho-S657+Y658 (Abcam Cat. #ab23513), antiglycogen synthase kinase 3 *α*/*β* antibody (GSK*α*/*β*) recognizing phospho-Y216+Y279 (Abcam Cat. #ab4797), the goat polyclonal antiaquaporin 1 (AQP1) antibody targeting the C-terminus of AQP1 of human origin (Santa Cruz Cat., Dallas, TX, #sc-9878), and the mouse monoclonal anti-MAP kinases ERK1/2 antibody recognizing phospho-Tyr 204 (Santa Cruz Cat. #sc-7383), respectively. The detailed analyses of these transporters or signaling proteins in the renal cortex have been described by us previously (Li and Zhuo 2011, 2013; Li et al. 2012a). Equal sample loading was normalized by treating the same membranes with stripping buffer (Pierce) for 20 min, blotting with 5% nonfat dry milk, and reprobing with a mouse anti-*β*-actin monoclonal antibody at 1:2000 (Sigma-Aldrich, St. Louis, MO). Western blot signals were analyzed using a Molecular Imager®, ChemiDoc™ XRS^+^ (Bio-Rad Laboratories, Inc., Hercules, CA). The changes in the expression of these proteins were normalized for comparisons using the ratio to *β*-actin protein.

### Data analysis and statistics

All data are presented as mean ± SEM. The differences between tg*Nhe3*^*+/+*^ and tg*Nhe3*^*−/−*^ mice in conscious systolic blood pressure, mean intra-arterial blood pressure, plasma aldosterone, plasma and kidney ANG II, and other intestinal and renal excretory responses were analyzed using Student’s unpaired *t*-test. The differences between basal responses and weekly responses to ANG II in the same group of tg*Nhe3*^*+/+*^ and tg*Nhe3*^*−/−*^ mice were first analyzed using one-way ANOVA followed by Dunnett’s comparisons between experimental treatments. All statistically different levels in or between responses were set *P *<* *0.05.

## Results

### Molecular phenotypes of tg*Nhe3*^*+/+*^ and tg*Nhe3*^*−/−*^ mice

**Figure 1:**
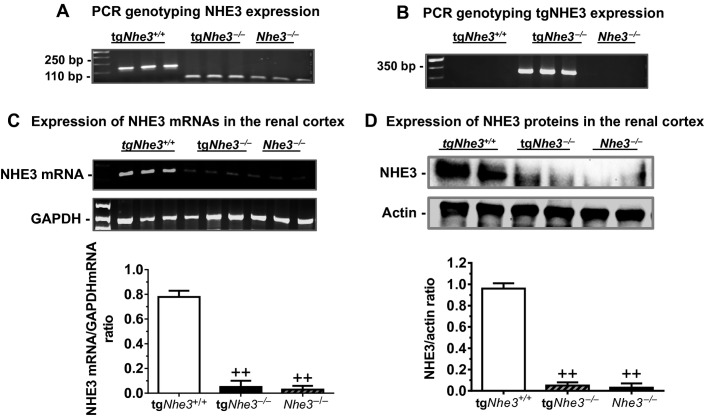
Molecular genotypes of wild-type *Nhe3*^*+/+*^ and tg*Nhe3*^*−/−*^ mice and comparisons with global *Nhe3*^*−/−*^ mice. (A) Southern blot genotyping of tg*Nhe3*^*+/+*^, tg*Nhe3*^*−/−*^, and global *Nhe3*^*−/−*^ mice, noting the lack of the *Nhe3* PCR product in both tg*Nhe3*^*−/−*^ and global *Nhe3*^*−/−*^ mice. (B) Southern blot genotyping of tg*Nhe3*^*−/−*^ mice in comparison with both tg*Nhe3*^*+/+*^ and *Nhe3*^*−/−*^ mice, noting the lack of the rescued *Nhe3* PCR product in both tg*Nhe3*^*+/+*^ and global *Nhe3*^*−/−*^ mice. Thus, tg*Nhe3*^*−/−*^ mice should show the lack of the *Nhe3* PCR product as *Nhe3*^*−/−*^ mice, but the expression of the rescued *Nhe3* transgene. (C) RT-PCR analysis of NHE3 mRNA expression in the superficial cortex of the kidney, primarily the proximal tubules, in tg*Nhe3*^*+/+*^ and tg*Nhe3*^*−/−*^ mice in comparisons with global *Nhe3*^*−/−*^ mice (*n* = 9). (D) Western blot analysis of NHE3 protein expression in the superficial cortex of the kidney, primarily the proximal tubules, in tg*Nhe3*^*+/+*^ and tg*Nhe3*^*−/−*^ mice in comparisons with global *Nhe3*^*−/−*^ mice (*n* = 6). >90% of NHE3 mRNA and protein expression were deleted in the proximal tubules of tg*Nhe3*^*−/−*^ and *Nhe3*^*−/−*^ mice. ^++^*P *<* *0.01 versus wild-type tg*Nhe3*^*+/+*^ mice.

The genotypes of all tg*Nhe3*^*+/+*^ and tg*Nhe3*^*−/−*^ mice used in this study were verified before the experiment was performed. Global *Nhe3*^*−/−*^ mice were also included for comparisons with tg*Nhe3*^*−/−*^ mice. In Figure1A, a representative Southern analysis of three tail DNA samples obtained from wild-type tg*Nhe3*^*+/+*^, tg*Nhe3*^*−/−*^ with transgenic rescue of the *Nhe3* gene in small intestines, and global *Nhe3*^*−/−*^ mice is shown for the presence or absence of the *Nhe3* gene. Both tg*Nhe3*^*−/−*^ and global *Nhe3*^*−/−*^ mice did not have the PCR product of wild-type tg*Nhe3*^*+/+*^ mice. The transgenic rescue of the *Nhe3* gene in small intestines of global *Nhe3*^*−/−*^ mice is further shown in Figure1B. The rescued *Nhe3* transgene was detected only in tg*Nhe3*^*−/−*^, but not in tg*Nhe3*^*+/+*^ or *Nhe3*^*−/−*^ mice. In further experiments, the expression of NHE3 mRNAs or NHE3 proteins in the superficial cortex of the kidney of tg*Nhe3*^*+/+*^, tg*Nhe3*^*−/−*^, and *Nhe3*^*−/−*^ mice was verified by semiquantitative RT-PCR (Fig.1C) or by western blot analysis (Fig.1D), respectively. Overall, more than 90–95% of NHE3 mRNAs and NHE3 proteins were deleted in the kidney of tg*Nhe3*^*−/−*^ mice, compared with tg*Nhe3*^*+/+*^ counterparts, therefore representing an alternative kidney-selective NHE3 knockout model (*n* ≥ 9 for each group).

### Morphological phenotypes of the digestive system in tg*Nhe3*^*+/+*^ and tg*Nhe3*^*−/−*^ mice

**Figure 2:**
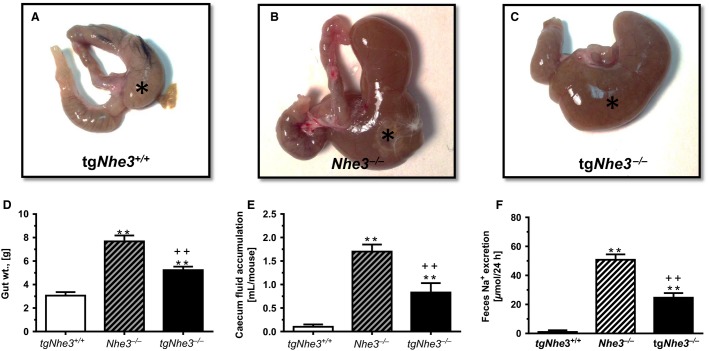
Comparisons of intestinal morphological and functional phenotypes of tg*Nhe3*^*+/+*^, tg*Nhe3*^*−/−*^, and *Nhe3*^*−/−*^ mice. Cecum segment between small and large intestines in (A) normal wild-type tg*Nhe3*^*+/+*^ mice (*), (B) global *Nhe3*^*−/−*^ mice, noting the extremely enlarged cecum segment accumulated with a large volume of fluid content inside (*), and (C) tg*Nhe3*^*−/−*^ mice with transgenic rescue of the Nhe3 gene in small intestines, noting improvements in the size and content in the cecum segment (*). Comparisons in the overall gut weight (D), fluid accumulation in the cecum segment (E), and 24 h fecal Na^+^ excretion (F) in tg*Nhe3*^*+/+*^ (*n* = 12), *Nhe3*^*−/−*^ (*n* = 15), and tg*Nhe3*^*−/−*^ (*n* = 15) mice. **P <* *0.01 versus tg*Nhe3*^*+/+*^. ^++^*P *<* *0.01 versus *Nhe3*^*−/−*^.

Despite transgenic rescue of the *Nhe3* gene primarily in small intestines of global *Nhe3*^*−/−*^ mice, mild to moderate diarrhea was still observed in tg*Nhe3*^*−/−*^ mice in the present study. About 20% of newly born homozygous tg*Nhe3*^*−/−*^ pups with the moderate to severe diarrhea phenotype still died during the first few weeks after birth, whereas the rest having the mild diarrhea phenotype were able to survive and grow normally to adulthood. There was no difference in body weight in age-matched male tg*Nhe3*^*+/+*^ and tg*Nhe3*^*−/−*^ mice (Table1). Compared with tg*Nhe3*^*+/+*^ mice, the overall weight of the digestive system more than doubled (Table1, *P* < 0.01), but it was significantly lower than that of global *Nhe3*^*−/−*^ mice (7.6 ± 0.5 g, *P *<* *0.01) (Fig.2). All segments of intestines of tg*Nhe3*^*−/−*^ mice appeared to be enlarged, compared with tg*Nhe3*^*+/+*^ mice, but showed significant improvement over global *Nhe3*^*−/−*^ mice (Fig.2). However, the most striking morphological abnormality was localized to the cecum segment between the small and large intestines of global *Nhe3*^*−/−*^ (Fig.2B) and tg*Nhe3*^*−/−*^ mice with transgenic rescue of the *Nhe3* gene (Fig.2C). Intestinal fluid accumulation in the cecum segment was significantly attenuated in tg*Nhe3*^*−/−*^ mice, compared with *Nhe3*^*−/−*^ mice (*Nhe3*^*−/−*^: 1.71 ± 0.15 mL vs. tg*Nhe3*^*−/−*^: 0.83 ± 0.20 mL, *P *<* *0.01) (Fig.2E). Fluid accumulation in the cecum segment of wild-type tg*Nhe3*^*+/+*^ mice was not observed. Similarly, 24 h fecal Na^+^ excretion rate was also significantly attenuated in tg*Nhe3*^*−/−*^ mice, compared with *Nhe3*^*−/−*^ mice (*Nhe3*^*−/−*^: 50.75 ± 3.78 *μ*mol/24 h vs. tg*Nhe3*^*−/−*^: 24.60 ± 3.20 *μ*mol/24 h, *P *<* *0.01) (Fig.2F). In wild-type tg*Nhe3*^*+/+*^ mice, 24 h fecal Na^+^ excretion was nearly negligible (1.2 ± 0.20 *μ*mol/24 h) (Fig.2F). No fluid accumulation was observed in the cecum segment of tg*Nhe3*^*+/+*^ mice.

**Table 1:** Basal cardiovascular and renal phenotypes of age-matched, male wild-type tg*Nhe3*^*+/+*^ and tg*Nhe3*^*−/−*^ mice with transgenic rescue of the *Nhe3* gene in small intestines

Parameter	tg*Nhe3*^*+/+*^ (*N* = 8–15)	tg*Nhe3*^*−/−*^ (*N* = 8–15)
Body wt., g	23.9 ± 0.2	24.7 ± 0.9
SBP, mmHg	121 ± 3	111 ± 3**
MAP, mmHg	93 ± 3	82 ± 3**
GFR, *μ*L/min	148.7 ± 13.0	83.9 ± 5.1**
Urine excretion, mL/24 h	1.25 ± 0.11	0.67 ± 0.08**
U_Na_V, *μ*mol/24 h	232.1 ± 10.3	30.7 ± 1.4**
U_K_V, *μ*mol/24 h	343.8 ± 19.4	180.5 ± 5.5**
U_Cl_V, *μ*mol/24 h	292.9 ± 19.4	219.7 ± 4.9**
H_2_O intake, mL/day	2.7 ± 0.4	5.0 ± 0.5**
Food intake, g/day	4.0 ± 0.4	5.8 ± 0.6**
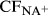 concentration, mmol/L	ND	97.9 ± 0.8
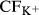 concentration, mmol/L	ND	33.8 ± 0.3
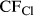 concentration, mmol/L	ND	37.8 ± 0.3
Heart wt. to body wt. ratio, ×100	0.49 ± 0.01	0.46 ± 0.01
Kidney wt. to body wt. ratio, ×100	1.21 ± 0.03	1.22 ± 0.03
Gut wt. to body wt. ratio, ×100	10.3 ± 0.2	23.9 ± 1.8**
Adrenal gland wt. to body wt. ratio, ×100	0.023 ± 0.002	0.043 ± 0.003**

SBP, systolic blood pressure under conscious conditions. MAP, mean arterial blood pressure under anesthesia. GFR, glomerular filtration rate, measured by the fluorescein isothiocyanate-inulin clearance technique (Li et al. 2009). U_Na_V, urinary Na^+^ excretion. U_K_V, urinary K^+^ excretion. U_Cl_V, urinary Cl^−^ excretion. 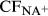, cecum fluid Na^+^ concentration. 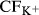, cecum fluid K^+^ concentration. 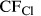, cecum fluid Cl^−^ concentration.

** *P *<* *0.01 versus tg*Nhe3*^*+/+*^ mice. ND, not determined because no fluid accumulation in the cecum segment of tg*Nhe3*^*+/+*^ mice.

### Morphological and basic excretory phenotypes of the kidney in tg*Nhe3*^*+/+*^ and tg*Nhe3*^*−/−*^ mice

**Figure 3:**
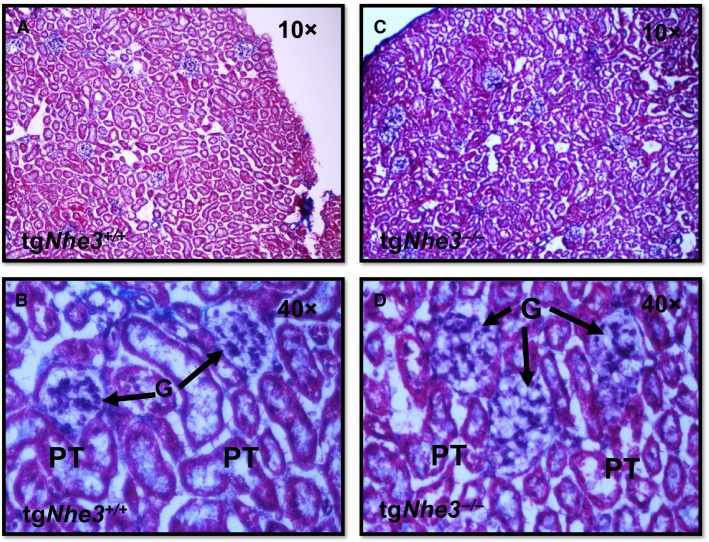
Comparisons of glomerular and proximal tubular histology between tg*Nhe3*^*+/+*^ and tg*Nhe3*^*−/−*^ mice with transgenic rescue of the *Nhe3* gene in small intestines. (A, B) Low (10×) and high (40×) magnification of the renal cortex of tg*Nhe3*^*+/+*^ mice. (C, D) Low (10×) and high (40×) magnification of the renal cortex of tg*Nhe3*^*−/−*^ mice. There were no significant differences in light microscopic glomerular and proximal tubule structures between tg*Nhe3*^*+/+*^ (*n* = 8) and tg*Nhe3*^*−/−*^ (*n* = 8) mice. G: glomerulus.

Upon visual inspection, the kidneys looked not significantly different between tg*Nhe3*^*−/−*^ mice and tg*Nhe3*^*+/+*^ mice, with similar kidney weight to body weight ratios (Table1). At the light microscopic level, the size and structure of the glomeruli were also similar between tg*Nhe3*^*−/−*^ and tg*Nhe3*^*+/+*^ mice (Fig.3). However, the wall of proximal tubules appeared to be slightly thinner and disorganized in tg*Nhe3*^*−/−*^ mice, compared with those of tg*Nhe3*^*+/+*^ mice (Fig.3). Although tg*Nhe3*^*−/−*^ mice drank and ate significantly more than tg*Nhe3*^*+/+*^ mice, GFR, 24 h urine excretion, urinary Na^+^ and K^+^ excretion rates were significantly lower in tg*Nhe3*^*−/−*^ mice (Table1).

### Basal blood pressure phenotype in tg*Nhe3*^*+/+*^ and tg*Nhe3*^*−/−*^ mice

In conscious age- and body weight-matched adult male tg*Nhe3*^*+/+*^ and tg*Nhe3*^*−/−*^ mice, basal systolic blood pressure, as determined by the tail-cuff method from five weekly measurements with at least 20 measurements each, was significantly lower in tg*Nhe3*^*−/−*^ than in tg*Nhe3*^*+/+*^ mice (*P *<* *0.01) (Table1). Mean intra-arterial blood pressure (MAP) was similarly lower in anesthetized tg*Nhe3*^*−/−*^ than tg*Nhe3*^*+/+*^ mice (*P *<* *0.01) (Table1).

### Basal plasma ANG II and plasma aldosterone levels and their responses to ANG II in tg*Nhe3*^*+/+*^ and tg*Nhe3*^*−/−*^ mice

**Figure 4:**
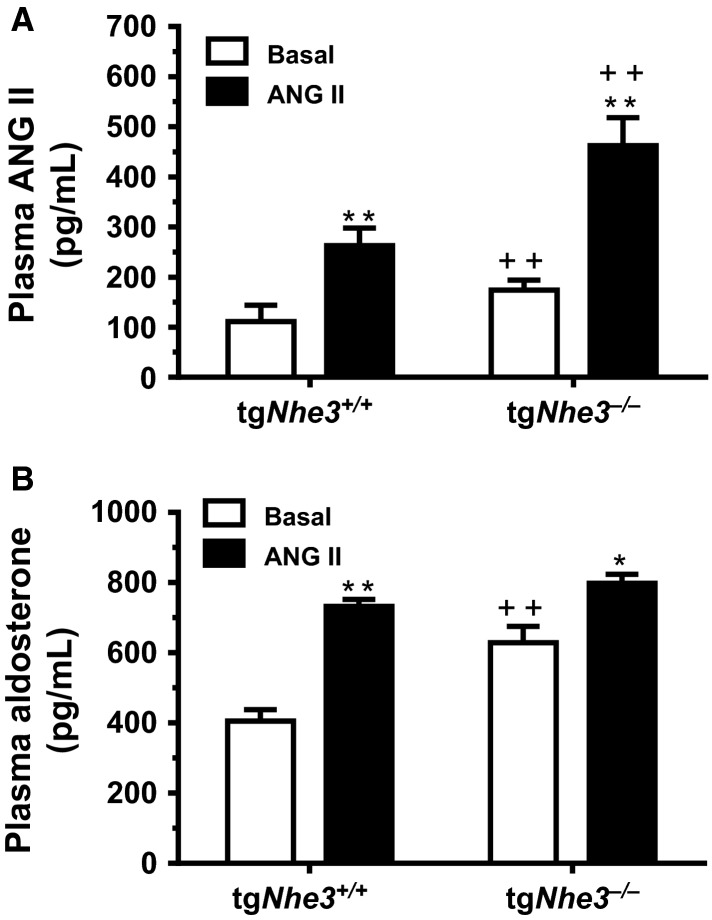
Comparisons of basal plasma ANG II and aldosterone levels under basal conditions and their responses to ANG II infusion (1.5 mg/kg/day, i.p., 2 weeks) in conscious tg*Nhe3*^*+/+*^ (*n* = 9) and tg*Nhe3*^*−/−*^ (*n* = 8) mice. (A) Plasma ANG II responses. (B) Plasma aldosterone responses. *P < 0.05 or **P <* *0.01 versus their basal levels in tg*Nhe3*^*+/+*^ or tg*Nhe3*^*−/−*^ mice. ^++^*P *<* *0.01 versus wild-type tg*Nhe3*^*+/+*^ mice under basal conditions or during ANG II infusion.

Basal plasma ANG II and aldosterone levels and their responses to ANG II infusion were determined to reveal the basal physiological status of the renin–angiotensin–aldosterone system (RAAS) underlying body salt and fluid volume homeostasis in tg*Nhe3*^*+/+*^ and tg*Nhe3*^*−/−*^ mice (Fig.4). Basal plasma ANG II level was significantly higher in tg*Nhe3*^*−/−*^ mice than wild-type mice (tg*Nhe3*^*+/+*^: 111 ± 33 pg/mL vs. tg*Nhe3*^*−/−*^: 174 ± 20 pg/mL, *P *<* *0.01) (Fig.4A). In responses to ANG II infusion, plasma ANG II increased to 263 ± 35 pg/mL in tg*Nhe3*^*+/+*^ mice (*P *<* *0.01) and to 462 ± 56 pg/mL in tg*Nhe3*^*−/−*^ mice (*P *<* *0.01), respectively (Fig.4A). Basal plasma aldosterone level was also significantly elevated in tg*Nhe3*^*−/−*^ mice (tg*Nhe3*^*+/+*^: 405 ± 33 pg/mL vs. tg*Nhe3*^*−/−*^: 629 ± 46 pg/mL, *P *<* *0.01) (Fig.4B). ANG II significantly increased plasma aldosterone level in tg*Nhe3*^*+/+*^ mice (*P *<* *0.01), but the response was attenuated in tg*Nhe3*^*−/−*^ mice (Fig.4B).

### Systolic and mean arterial pressure responses to ANG II in conscious and anesthetized tg*Nhe3*^*+/+*^ and tg*Nhe3*^*−/−*^ mice

**Figure 5:**
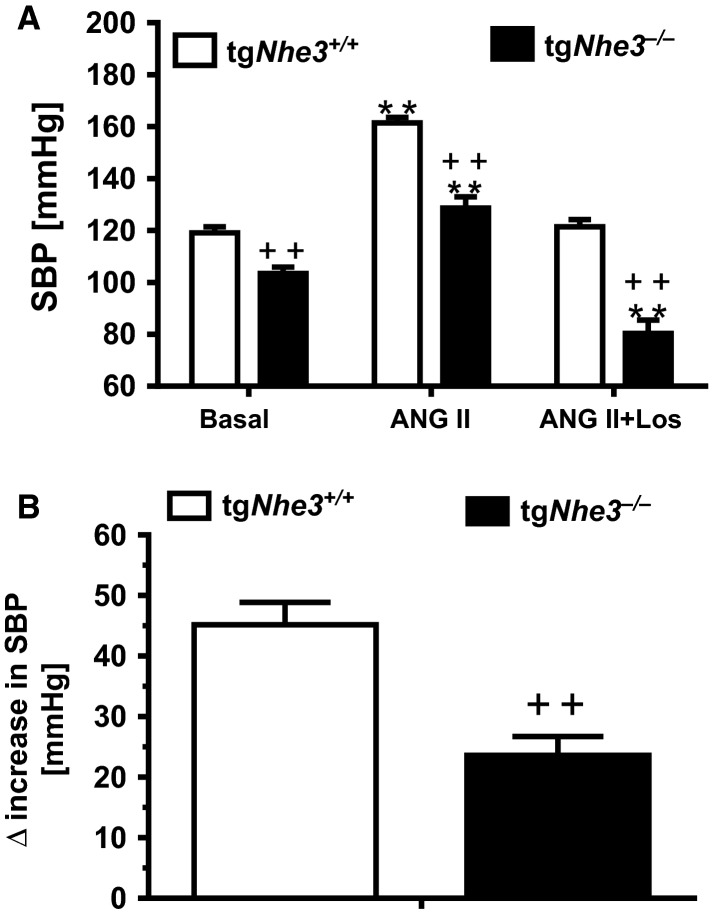
Comparisons of basal systolic blood pressure (SBP) and its responses to ANG II in conscious adult male tg*Nhe3*^*+/+*^ and tg*Nhe3*^*−/−*^ mice. (A) Note that basal SBP were significantly lower in conscious tg*Nhe3*^*−/−*^ mice (*n* = 12) than tg*Nhe3*^*+/+*^ mice (*n* = 15), and that SBP responses to ANG II (1.5 mg/kg/day, i.p., 2 weeks) were markedly attenuated in tg*Nhe3*^*−/−*^ mice. **P <* *0.01 versus their basal SBP. ^++^*P *<* *0.01 versus tg*Nhe3*^*+/+*^ during the same treatment. Losartan completely blocked ANG II-increased SBP in tg*Nhe3*^*+/+*^ mice (*n* = 8), but markedly reduced SBP significantly below their basal SBP in tg*Nhe3*^*−/−*^ mice (*n* = 6). (B) Net increase in SBP in response to ANG II in tg*Nhe3*^*+/+*^ and tg*Nhe3*^*−/−*^ mice, respectively.

**Figure 6:**
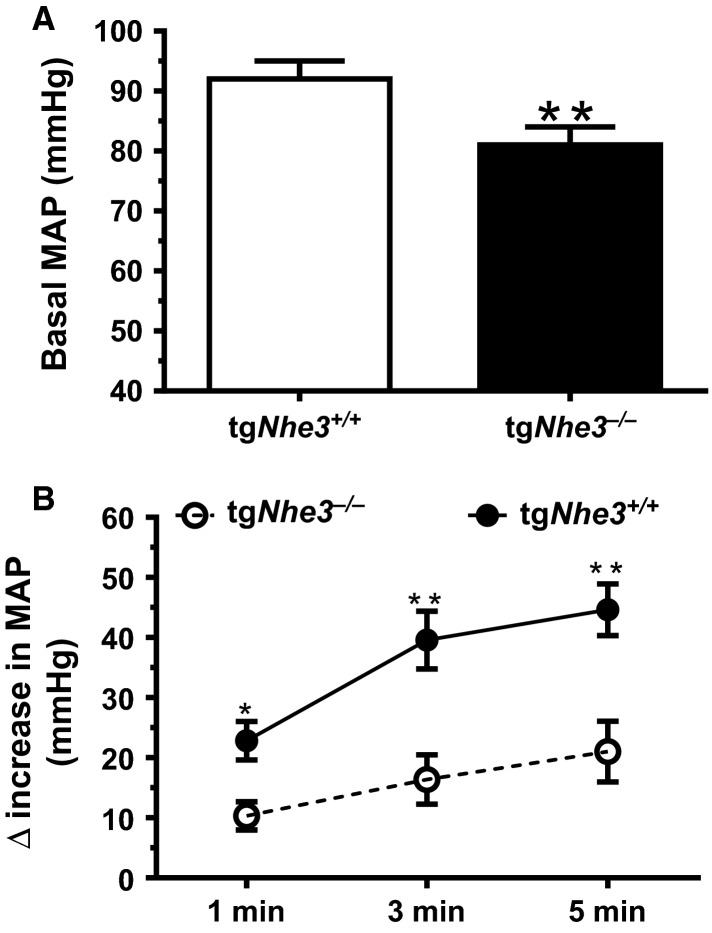
Comparisons of basal mean intra-arterial blood pressure (MAP) and its responses to acute ANG II infusion, 10 pmol/min, i.v., in anesthetized tg*Nhe3*^*+/+*^ (*n* = 7) and tg*Nhe3*^*−/−*^ (*n* = 8) mice. (A) Basal MAP was significantly lower in tg*Nhe3*^*−/−*^ mice (**P <* *0.01). (B) MAP responses to ANG II were significantly attenuated in tg*Nhe3*^*−/−*^ mice. **P <* *0.01 versus tg*Nhe3*^*−/−*^ mice at the same time point.

Systolic (SBP) and mean intra-arterial pressure (MAP) responses to ANG II stimulation were significantly different in both conscious (Fig.5) and anesthetized tg*Nhe3*^*+/+*^ and tg*Nhe3*^*−/−*^ mice (Fig.6). In response to a high pressor dose of ANG II infusion (1.5 mg/kg/day, i.p.), SBP was increased by 48 ± 3 mmHg in conscious tg*Nhe3*^*+/+*^ mice from its baseline level (*P *<* *0.01, Fig.5). By contrast, SBP was increased by only 19 ± 3 mmHg in conscious tg*Nhe3*^*−/−*^ mice from its baseline level (*P *<* *0.01, Fig.5). The pressor responses to ANG II were blocked by concurrent treatment with the AT_1_ receptor blocker losartan in tg*Nhe3*^*+/+*^ mice, but losartan markedly decreased SBP responses to ANG II in tg*Nhe3*^*−/−*^ mice significantly below the basal levels (Fig.5A). Under the anesthetic condition (Fig.6), peak MAP was increased by ANG II by an average of 46 ± 3 mmHg in anesthetized tg*Nhe3*^*+/+*^ mice at 5 min (*P *<* *0.01). By comparison, peak MAP was increased by ANG II by an average of 25 ± 3 mmHg in anesthetized tg*Nhe3*^*−/−*^ mice at 5 min (*P *<* *0.01). The differences in MAP responses to ANG II lasted throughout the duration of ANG II infusion between tg*Nhe3*^*+/+*^ and tg*Nhe3*^*−/−*^ mice (*P *<* *0.01, Fig.6). Furthermore, anesthetized tg*Nhe3*^*−/−*^ mice were unable to tolerate the AT_1_ receptor blockade with losartan since MAP fell rapidly, so that the experiments were unable to continue in these mice.

### Basal proximal tubule transporter protein expression in tg*Nhe3*^*+/+*^ and tg*Nhe3*^*−/−*^ mice

**Figure 7:**
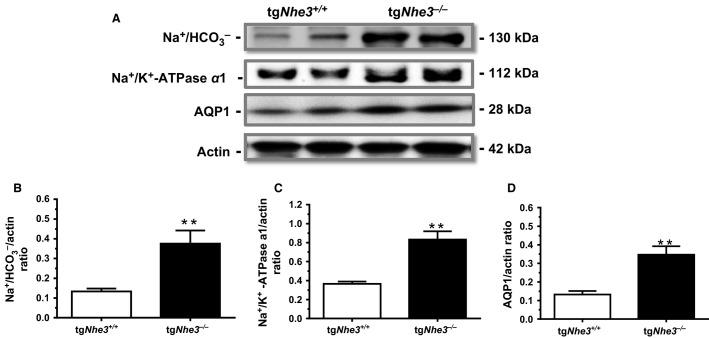
Comparisons of the expression of major water channel and sodium transporter proteins in the superficial cortex of the kidney, primarily the proximal tubules, in tg*Nhe3*^*+/+*^ and tg*Nhe3*^*−/−*^ mice (*n* = 6 for each strain) under basal conditions. AQP, aquaporin 1. Na^+^/HCO_3_^−^, the sodium and bicarbonate cotransporter. Na^+^/K^+^-ATPase *α*1, the sodium and potassium ATPase *α*1 isoform. (A) Representative Western blots of selective transporter proteins. (B) Semi-quantitated data on the sodium and bicarbonate cotransporter. (C) Semi-quantitated data on the sodium and potassium ATPase α1 isoform. (D) Semi-quantitated data on Aquaporin 1. **P <* *0.01 versus tg*Nhe3*^*+/+*^ mice.

The expression of three major representative transporter proteins in the proximal tubules of the superficial renal cortex was measured at the basal level to determine the compensatory mechanisms in tg*Nhe3*^*−/−*^ mice (Fig.7). Under basal conditions, the expression of the Na^+^/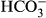 cotransporter proteins was significantly upregulated in tg*Nhe3*^*−/−*^ mice (tg*Nhe3*^*+/+*^: 0.13 ± 0.03 vs. tg*Nhe3*^*−/−*^: 0.38 ± 0.07 Na^+^/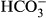/actin ratio, *P *<* *0.01). The expression of the Na^+^/K^+^-ATPase *α*1 subunit proteins was also significantly upregulated in tg*Nhe3*^*−/−*^ mice (tg*Nhe3*^*+/+*^: 0.37 ± 0.03 vs. tg*Nhe3*^*−/−*^: 0.83 ± 0.09 Na^+^/K^+^-ATPase/actin ratio, *P *<* *0.01). Similarly, the expression of the major water channel aquaporin 1 (AQP1) proteins in the proximal tubules was significantly upregulated in tg*Nhe3*^*−/−*^ mice (tg*Nhe3*^*+/+*^: 0.13 ± 0.02 vs. tg*Nhe3*^*−/−*^: 0.35 ± 0.05 AQP1/actin ratio, *P *<* *0.01) (Fig.7).

### Proximal tubule transporter protein responses to ANG II in tg*Nhe3*^*+/+*^ and tg*Nhe3*^*−/−*^ mice

**Figure 8:**
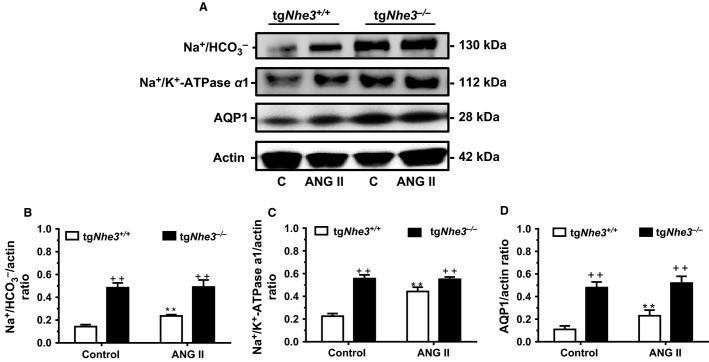
Comparisons of the responses to ANG II (1.5 mg/kg/day, i.p., 2 weeks) in the expression of Na^+^/HCO_3_^−^, Na^+^/K^+^-ATPase *α*1, and AQP1 proteins in the superficial cortex of the kidney in tg*Nhe3*^*+/+*^ and tg*Nhe3*^*−/−*^ mice (*n* = 6 for each strain). (A) Representative Western blots of selective transporter protein responses to ANG II. (B) Semi-quantitated data on the sodium and bicarbonate cotransporter. (C) Semi-quantitated data on the sodium and potassium ATPase α1 isoform. (D) Semi-quantitated data on Aquaporin 1. **P <* *0.01 versus basal control tg*Nhe3*^*+/+*^ mice. ^++^*P *<* *0.01 versus control or ANG II-infused tg*Nhe3*^*+/+*^ mice. Note the complete lack of responses to ANG II in tg*Nhe3*^*−/−*^ mice.

In response to ANG II infusion, the expression of the Na^+^/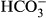 cotransporter proteins was significantly stimulated in tg*Nhe3*^*+/+*^ mice (tg*Nhe3*^*+/+*^: 0.19 ± 0.05 vs. tg*Nhe3*^*−/−*^: 0.49 ± 0.05 Na^+^/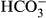/actin ratio, *P *<* *0.01) (Fig.8). Similarly, The expression of the Na^+^/K^+^-ATPase *α*1 subunit proteins was also significantly stimulated by ANG II in tg*Nhe3*^*+/+*^ mice (tg*Nhe3*^*+/+*^: 0.43 ± 0.11 vs. tg*Nhe3*^*−/−*^: 0.55 ± 0.10 Na^+^/K^+^-ATPase *α*1/actin ratio, *P *<* *0.01) (Fig.8). Likewise, AQP proteins in tg*Nhe3*^*+/+*^ mice were moderately increased by ANG II (tg*Nhe3*^*+/+*^: 0.17 ± 0.06 vs. tg*Nhe3*^*−/−*^: 0.50 ± 0.02 AQP1/actin ratio, *P *<* *0.01). However, neither the Na^+^/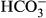 nor the Na^+^/K^+^-ATPase *α*1 or AQP1 protein expression was responded to ANG II stimulation in tg*Nhe3*^*−/−*^ mice (Fig.8).

### Proximal tubule signaling protein responses to ANG II in tg*Nhe3*^*+/+*^ and tg*Nhe3*^*−/−*^ mice

**Figure 9:**
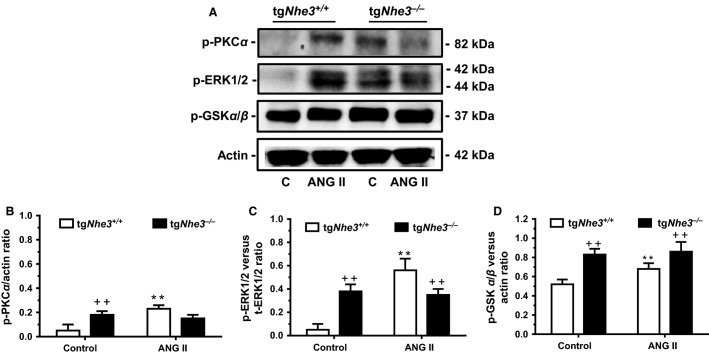
Comparisons of major signaling protein levels and their responses to ANG II (1.5 mg/kg/day, i.p., 2 weeks) in the renal cortex, primarily in the proximal tubules of tg*Nhe3*^*+/+*^ and tg*Nhe3*^*−/−*^ mice. (A) Representative Western blots of selective signaling protein responses. Note that under basal conditions, phosphorylated protein kinase C*α* isoform (B, p-PKC*α*), MAP kinases ERK1/2 (C, p-ERK1/2), and glycogen synthase kinase 3*α*/*β* (D, p-GSK3*α*/*β*) protein expression were all increased in the superficial cortex of the kidney, primarily the proximal tubules in tg*Nhe3*^*+/+*^ mice (*n* = 6 for each strain). While these signaling proteins were significantly increased by ANG II in tg*Nhe3*^*+/+*^ mice, there were no differences in these signaling proteins in tg*Nhe3*^*−/−*^ mice with or without ANG II infusion. **P <* *0.01 versus basal control tg*Nhe3*^*+/+*^ mice. ^++^*P *<* *0.01 versus basal control or ANG II-infused tg*Nhe3*^*+/+*^ mice.

In wild-type tg*Nhe3*^*+/+*^ mice, infusion of ANG II for 2 weeks significantly increased phosphorylated protein kinase C*α* (PKC*α*) levels (Control: 0.03 ± 0.03 vs. ANG II: 0.23 ± 0.05 p-PKC*α*/actin ratio, *P *<* *0.01), and phosphorylated MAP kinases ERK1/2 (Control: 0.05 ± 0.03 vs. ANG II: 0.56 ± 0.10 p-ERK1/2/actin ratio, *P *<* *0.01) (Fig.9). Phosphorylated glycogen synthase kinase 3*α*/*β* was also significantly elevated. By comparison, there were no significances in all three signaling protein expression under basal condition and in response to ANG II infusion for 2 weeks in tg*Nhe3*^*−/−*^ mice (Fig.9).

## Discussion

Despite extensive investigations over several decades, the mechanisms of ANG II-induced or ANG II-dependent hypertension remain incompletely understood. Although the involvement of ANG II-induced increases in sympathetic neural activity, cardiac hypertrophy, and vascular constriction, renal vasoconstriction and salt retention has been extensively investigated, their respective contributions remain difficult to be defined. Recently, we investigated the role of global NHE3 in ANG II-induced hypertension using mutant mice with global deletion of NHE3 in target tissues including the kidney and the digestive system, *Nhe3*^*−/−*^ mice (Li et al. 2015). The results of that particular study demonstrated two key findings with potentially physiological as well as clinical implications. The first conclusion is that NHE3 is absolutely necessary for maintaining basal blood pressure homeostasis due to its physiological action on promoting Na^+^ absorption from the small intestines and Na^+^ reabsorption from the proximal tubule of the kidney. Genetic removal of NHE3 in all tissues led to a decrease in basal blood pressure by ∼13 ± 3 mmHg (Schultheis et al. 1998; Li et al. 2015). The second conclusion is that NHE3 is also required for the full development of ANG II-dependent hypertension. That conclusion is supported by the findings that ANG II-induced hypertension was significantly attenuated in global *Nhe3*^*−/−*^ mice. Thus, those results strongly suggest that NHE3 indeed plays a key role in the physiological regulation of blood pressure, and is involved in the full development of ANG II-induced hypertension. However, limitations of that study are that global *Nhe3*^*−/−*^ mice were used to test the hypothesis. All *Nhe3*^*−/−*^ mice had moderate to severe intestinal phenotypes including diarrhea and fluid accumulation in the cecum segment between small and large intestines, resulting in marked salt wasting from the intestines (Schultheis et al. 1998; Li et al. 2015) and compensatory salt retention from the kidney (Lorenz et al. 1999; Wang et al. 1999; Li et al. 2015). Because *Nhe3*^*−/−*^ mice are a global NHE3 knockout model, previous studies were unable to separate the contributions between intestinal versus kidney NHE3 in blood pressure responses to endogenous or exogenous ANG II. To further test our hypothesis, we used a better genetic mouse model, namely global *Nhe3*^*−/−*^ mice with transgenic rescue of the *Nhe3* gene selectively in small intestines, tg*Nhe3*^*−/−*^*,* using the intestinal fatty acid binding protein (IFABP) promoter in the present study (Woo et al. 2003). The rationale is that there is very little NHE3 expression beyond the small intestines of the digestive system and the kidney, so that tg*Nhe3*^*−/−*^ mice may be considered alternatively to be the kidney-selective NHE3 knockout mice (Woo et al. 2003; Noonan et al. 2005).

The key objective of the current study was not to evaluate basic intestinal and renal phenotypes such as the body salt and fluid status and tolerance of high salt intake of tg*Nhe3*^*−/−*^ mice as reported previously by Woo et al. (2003) and Noonan et al. (2005). Instead, we specifically determined whether the development of ANG II-induced hypertension would be attenuated in tg*Nhe3*^*−/−*^ mice with transgenic rescue of the NHE3 gene selectively in small intestines. The hypothesis tested was that NHE3 in the kidney plays a key role in ANG II-induced hypertensive responses. A direct interaction or relationship between ANG II and NHE3 in the kidney has been studied previously in cultured proximal tubule cells or the proximal tubules of the kidney. We and others have previously showed that ANG II significantly increases NHE3 expression and activity in cultured proximal tubule cells (Geibel et al. 1990; du et al. 2003; Li and Zhuo 2008b; Li et al. 2012a) or in the proximal tubule of the kidney (Banday and Lokhandwala 2011; Li and Zhuo 2011, 2013). NHE3 is the most important Na^+^ transporter in apical membranes of the proximal tubules (Lorenz et al. 1999; Wang et al. 1999; Vallon et al. 2000; McDonough 2010). Indeed, NHE3 not only directly contributes to about 25% of Na^+^ reabsorption (Wilcox et al. 1992; McDonough 2010), but also acts indirectly to drive passive reabsorption of additional 50% of the filtered Na^+^ load in the proximal tubules of the kidney (Rector 1983; Schafer and Robert 1984; Berry and Rector 1991). Hypertensive effects of nonpressor or slow pressor doses, but not acute high pressor doses, of ANG II are mediated at least in part by stimulating NHE3 expression/activity in the proximal tubules of the kidney (Banday and Lokhandwala 2011; Li and Zhuo 2011, 2013). The results of the present study clearly confirmed our hypothesis by demonstrating that both systolic blood pressure responses to ANG II in conscious tg*Nhe3*^*−/−*^ mice (Fig.5) and intra-arterial MAP responses to ANG II in anesthetized tg*Nhe3*^*−/−*^ mice (Fig.6) were markedly attenuated.

Although the mechanisms underlying these attenuated hypertensive responses to ANG II in tg*Nhe3*^*−/−*^ mice were not fully investigated in the present study, some plausible mechanisms were still uncovered. The roles of nonrenal and nonintestinal NHE3 may probably be minimal, because there is little evidence that a significant level of NHE3 is expressed in the blood vessels, the heart, the adrenal glands, or the brain. Since the functional *Nhe3* gene was in theory replaced in small intestines of tg*Nhe3*^*−/−*^ mice (Woo et al. 2003; Noonan et al. 2005), and NHE3 is primarily expressed in small intestines in the digestive system, our results may at least in part be attributed to the absence of NHE3 expression and function in the proximal tubule of the kidney in these mice. Indeed, only a small fraction of NHE3 is reportedly expressed in renal tubules beyond the proximal tubules of the kidney, primarily in the loop of Henle (Amemiya et al. 1995; Biemesderfer et al. 1997). The impaired or attenuated ANG II responses in tg*Nhe3*^*−/−*^ mice was unlikely to relate to structural abnormalities in the kidney, because tg*Nhe3*^*−/−*^ mice show basically similar glomerular and proximal tubular structures of wild-type animals (Fig.3). By contrast, we recently found that the vascular pole of numerous glomeruli became enlarged or hypertrophic in global *Nhe3*^*−/−*^ mice, consistent with increased expression of kidney renin (Schultheis et al. 1998) and kidney ANG II levels (Li et al. 2015), and reportedly significantly reduced GFR (Ledoussal et al. 2001). However, the vascular pole of the glomeruli in tg*Nhe3*^*−/−*^ mice looks similar to wild-type counterparts in the present study (Fig.3). The third potential mechanism may be due to the incomplete rescue of the *Nhe3* gene expression and function in small intestines of tg*Nhe3*^*−/−*^ mice. The results of the present study largely replicated the basic intestinal phenotypes of these mice reported previously (Woo et al. 2003; Noonan et al. 2005). Although significantly improved from global *Nhe3*^*−/−*^ mice, small intestines of tg*Nhe3*^*−/−*^ mice remained enlarged relative to tg*Nhe3*^*+/+*^ mice, the entire digestive system was still significantly heavier, a large volume of fluid was still accumulated in the cecum segment, whereas 24 h Na^+^ excretion from the feces was still significantly increased in tg*Nhe3*^*−/−*^ mice (Fig.2). These results suggest that the rescue of the *Nhe3* gene in small intestines of these mice may be incomplete. This may have led to continuous and significant salt wasting from the digestive system and subsequent causing body salt and fluid contraction in tg*Nhe3*^*−/−*^ mice.

The most important mechanism underlying the attenuated hypertensive responses to ANG II in tg*Nhe3*^*−/−*^ mice is most likely that the entire circulating and tissue renin–angiotensin–aldosterone system (RAAS) is markedly activated in response to salt wasting from the gut in tg*Nhe3*^*−/−*^ mice. Indeed, basal plasma ANG II and aldosterone levels were significantly elevated in these mice in the present study (Fig.4). Our results provide further support to previous studies in which renal renin and serum aldosterone levels were markedly elevated in global *Nhe3*^*−/−*^ (Schultheis et al. 1998; Li et al. 2015) and tg*Nhe3*^*−/−*^ mice (Woo et al. 2003). Although the expression of AT_1_ receptors in the kidney of tg*Nhe3*^*−/−*^ mice was previously found to be similar to that in wild-type mice (Noonan et al. 2005), these receptors in target tissues may be theoretically downregulated by high circulating ANG II. Alternatively, AT_1_ receptors may be desensitized by high plasma ANG II, so that the hypertensive effects of ANG II were significantly diminished in tg*Nhe3*^*−/−*^ mice (Figs.5 and 6). Consistent with these interpretations are that under basal conditions with high plasma ANG II and aldosterone levels, the expression of several key water (AQP1) and Na^+^ transporter or cotransporter proteins (Na^+^/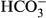 and the Na^+^/K^+^-ATPase *α*1), as well as some key signaling proteins (p-PKC*α*, p-ERK1/2, and p-GSK3*α*/*β*), in the proximal tubules of the kidney was markedly upregulated (Figs. 7–9). While the expression of all of these transporter or signaling proteins was responded robustly to ANG II stimulation in wild-type animals, these responses were all lost or attenuated in tg*Nhe3*^*−/−*^ mice.

Taken together, the results of the present study strongly support the overall hypothesis that NHE3 is necessary for maintaining basal blood pressure homoeostasis and the full development of ANG II-dependent hypertension. However, it should be recognized that, despite its phenotypic and functional improvements from global *Nhe3*^*−/−*^ mice, our results suggest that tg*Nhe3*^*−/−*^ mice remain to be an inadequate mutant mouse model to determine the relative role or contribution of proximal tubule or kidney NHE3 in the physiological regulation of blood pressure and the development of ANG II-dependent hypertension. Major renal functional phenotypes of global *Nhe3*^*−/−*^ mice persist in tg*Nhe3*^*−/−*^ mice due to the incomplete rescue of the *Nhe3* gene in the digestive system (Woo et al. 2003; Noonan et al. 2005). As salt wasting continues to occur in the gut, the kidney responds to the loss of NHE3, and subsequent activation of the RAAS and/or other salt-retaining peptides or hormones to mobilize other water and Na^+^ transporters or cotransporters in the proximal and distal nephron segments. The marked decreases in basal GFR, 24 h urine excretion, and urinary Na^+^ excretion observed in tg*Nhe3*^*−/−*^ mice (Table1) are consistent with this interpretation. Thus, a newer rodent model with conditional knockout of the *Nhe3* gene selectively in the intestines or in the proximal tubules of the kidney using the Cre/LoxP approach (Li et al. 2013) may be necessary to further dissect the specific role of intestinal versus proximal tubule NHE3 in blood pressure regulation and the development of ANG II-dependent or independent hypertension.

## Conflict of Interest

None declared.
